# Organon Society of Boston, Regular Meeting

**Published:** 1888-06

**Authors:** S. A. Kimball

**Affiliations:** Secretary; Boston


### REGULAR MEETING ORGANON SOCIETY.

Boston, April 19th, 1888.

Dr. Bell read from the *Organon* beginning at Section 14, Dr. Wesselhœft being absent.

Dr. Bell—In regard to the 16th section, I saw in a magazine a short time since that cancer was a disease of the nerves, because the nerves presided over nutrition, and cancer was the result of mal-nutrition, hence cancer was a disease of the nerves.

The same can be said of any disease.

The foot-note to the 17th section, note 8, quite well illustrates the condition of the mind cure in regard to what it can do.

Dr. Kennedy—I would like to ask what is meant by "the entire complex of symptoms" in Sec. 17?

Dr. Bell—I think it means the totality of the symptoms, the disease. You might except latent syphilis, perhaps, when there are no external manifestations of the disease.

Dr. Kennedy—We are often told that if a skin disease is treated by external application, and it disappears, the patient is well, and oftentimes he seems well, and says he feels perfectly well.

Dr. Bell—I think all such patients would show signs of psora if closely questioned according to the list of psoric symptoms in the first volume of the *Chronic Diseases.*

Dr. Kennedy—We are also often told that a skin disease is a skin disease and nothing else.

Dr. Bell—There is one answer that can be made to that statement. Ask them what syphilis is, if that is simply a skin disease. It was thought to be by the allopaths until they began to find out its effects upon the internal organs.

Dr. Hastings—They would probably say that psora cannot be traced, as syphilis can.

Dr. Bell—Psora can be traced in the same way exactly, and just as clearly. I wish to relate a case I heard of in the Highlands the other day that was most shamefully managed. A servant girl was sick. An allopathic physician was called and injected a quarter of a grain of Morphine. The next day she was no better, and a homœopathic (?) physician was called. He gave *half* a grain of Morphine in the morning and *half* a grain at night.

The next day, strange to say, she was no better, and the same thing was done again. It can scarcely be called treatment—half a grain in the morning and half a grain in the evening. The next day the patient was dead. The disease was peritonitis, but the cause of death was only too evident.

Even the allopathic physician said that no man should give a half a grain of Morphine without remaining with his patient, which was not done in this case. I would now like to read the 18th section:

"It is then unquestionably true that, besides the totality of symptoms, it is impossible to discover any other manifestation by which diseases could express their need of relief. Hence, it undoubtedly follows that the totality of symptoms observed in each individual case of disease can be the *only indication* to guide us in the selection of a remedy."

Now I have been told that a prominent so-called homœopathic oculist had stated to his class that they would not be able to cure their eye cases with the indicated remedy; that the indicated remedy was "all bosh."

In opposition to this statement, and in confirmation of the truth of the 18th section, Dr. F. W. Payne has a very interesting case to give us.

Dr. Payne then read a case of retinitis pigmentosa, which was much enjoyed by those present. The maps showing the increase of sight were particularly interesting, as they were solid facts, showing a gradual increase of vision that could not be disputed. (This case is given in full on another page.)

Dr. Jameson—I wish Dr. John Payne would relate a very interesting eye case that he told me the other day.

Dr. J. H. Payne—There is very little to say about the case. I prescribed on one symptom alone, as that seemed to be the only one in the case. The patient, a woman thirty-five years of age, was brought to my office for consultation. There was a deep scar of the cornea resulting from ulceration when an infant, and in that eye there was scarcely a perception of sight, nor had there been for years. Otherwise she seemed perfectly well. I advised Kali bich. on account of the symptom, from Hering, I think, "deep scar in the cornea following an ulcerative condition."

In two or three months her physician again brought her in with the cornea perfectly clear and sight completely restored. I think he used the 30th, giving it for several days and then stopping it.

I would like to ask Dr. Bell how often he would repeat a remedy.

Dr. Bell—Only when the case ceases to improve, and be sure it is ceasing to improve.

Dr. Winn—How often would you repeat in acute cases—gall-stone colic, for instance?

Dr. Bell—In such cases I usually give one dose dry and then wait; if there is no improvement in fifteen, twenty, or thirty minutes, I give another remedy, also dry, but never repeat the same remedy unless there has been an improvement in that time, and then, if the improvement ceases, the same remedy may be repeated. Usually, however, one dose of the right remedy is sufficient.

Dr. Winn—I have had a case of gall-stone colic recently with pain in right side of abdomen through to back, radiating from the right abdomen to chest and shoulder, with exquisite tenderness to touch, and sensitiveness to jar. The first one or two remedies given did not relieve, and on further questioning it was found that there was ringing in ears, desire to belch, thirst, coldness of surface, and much exhaustion. China$^{200}$ was given in water, and the effect of the first dose was almost magical, and the relief was immediate. Another paroxysm came on in fifteen minutes, and the dose was repeated, then not again for an hour, and after that once or twice at intervals of three or four hours; but every time the pain returned the China controlled it. The next day she was all right but sore.

Dr. Bell—The 20th section calls to my mind the fact that the appearance of the medicine not infrequently has considerable effect on patients. They will say, "Doctor, how can such a small, white powder have any effect?" I usually ask them if they ever saw a grain of Arsenic or Tartar Emetic, and tell them that it looks exactly like the sugar powder.

Dr. Winn—Do we not often get a group of symptoms that seem perfectly covered by a remedy and yet we get no result?

Dr. Bell—I think that we often prescribe with the greatest care and feel very confident of a cure, yet we do not cure, and often the remedy seems to have no effect. We see this especially in hypochondriacs, very nervous patients, etc. The symptoms often change; the next time they come we get an entirely different picture. Such patients are morbidly organized, they can be greatly helped but not thoroughly cured.

Dr. Payne—Is not latent psora at the bottom of such cases?

Dr. Bell—They seem to be so deeply psoric that the vital force does not seem to react to the remedy.

Adjourned to May 3d.

S. A. KIMBALL, *Secretary.*

